# Supplementary material for: The national burden from O’nyong’nyong and chikungunya viruses in Cameroon
Source: medRxiv. 2026 Jul 1:2026.06.24.26356335. Preprint. [Version 1] doi: 10.64898/2026.06.24.26356335 (PMC13345575; doi:10.64898/2026.06.24.26356335)
Supplement: Supplementary file 1 [file NIHPP2026.06.24.26356335V1-supplement-1.pdf]

medRxiv preprint doi: https://doi.org/10.64898/2026.06.24.26356335; this version posted July 1, 2026. The copyright holder for this preprint
(which was not certified by peer review) is the author/funder, who has granted medRxiv a license to display the preprint in perpetuity.
It is made available under a CC-BY 4.0 International license .

Supplementary materials

Parameter	Parameter description	Prior
π	Prevalence	beta (1,5)
μ0	Mean of negative titres	normal (5,0.5)
μ1	Mean increase of positive tires	normal (2.5,0.1)
σ0	SD of negative titres	normal (1,0.1)
σ1	SD of positive tires	normal (0.3, 0.05)
ϕ	Cross reactivity	lognormal (log (0.3,0.3)
ρ	Correlation of negative titres	beta (3,7)

Table 1: Multivariate mixture model parameter priors.
Parameter priors and limits used in model fitting.

medRxiv preprint doi: https://doi.org/10.64898/2026.06.24.26356335; this version posted July 1, 2026. The copyright holder for this preprint (which was not certified by peer review) is the author/funder, who has granted medRxiv a license to display the preprint in perpetuity.
It is made available under a CC-BY 4.0 International license .

For Model 1, which assumes the presence of ONNV and CHIK (and absence of MAYV), 920 samples with missing CHIK data were excluded. To assess the impact of this exclusion, we fitted two versions of Model 2: one using only ONNV titre data, and one incorporating the full dataset, including the 920 samples missing CHIK measurements but complete for ONNV and MAYV. Median parameter estimates were broadly comparable across both versions, and spatially varying FOI estimates were derived for each.

**Multivariate Mixture Model Parameter estimates**		
Parameter	**Model 1 (Full Model: ONNV +ve, CHIKV +ve)**	**Model 2 (ONNV Only Model)**
π_{ONNV}	0.23 (95%CI:0.22 - 0.25)	0.23 (95%CI:0.21 - 0.24)
$\mu0_{ONNV}$	4.54 (95%CI: 4.51 - 4.57)	4.64 (95%CI: 4.61 - 4.67)
$\mu1_{ONNV}$	6.57 (95%CI: 6.54 - 6.60)	6.65 (95%CI: 6.62 - 6.68)
$\phi_{ONNV \rightarrow MAYV}$	0.97(95%CI: 0.92 - 1.0)	1.0 (95%CI: 0.97 -1.05)
**Spatially varying FOI**		
	**Model 1 (Full Model: ONNV +ve, CHIKV +ve)**	**Model 2 (ONNV Only Model)**
FOI	0.018 (95% CI: 0.015 - 0.02)	0.017 (95% CI: 0.014 - 0.02)

Table 2: Comparison of the Full model (ONNV and CHIKV samples) with the ONNV only model (including the 920 samples missing CHIKV measurements).
Median parameter estimates with 95% CI and FOI estimates for both models.

medRxiv preprint doi: https://doi.org/10.64898/2026.06.24.26356335; this version posted July 1, 2026. The copyright holder for this preprint (which was not certified by peer review) is the author/funder, who has granted medRxiv a license to display the preprint in perpetuity.
It is made available under a CC-BY 4.0 International license .

Region	Region Population	Population Weighted FOI estimate	Population Weighted seropositivity estimate	Annual Infections
Adamawa	1434657	0.0197	0.32	17233
Centre	5450965	0.0194	0.32	71826
East	1207469	0.022	0.35	14327
Far North	4608223	0.013	0.24	43315
Littoral	4073615	0.018	0.30	49880
North	3082097	0.016	0.28	32889
North-West	2570428	0.011	0.199	18295
West	2046142	0.012	0.22	18475
South	1154882	0.021	0.34	12716
South-West	2087437	0.019	0.31	23092

Table 3: FOI, Seropositivity and Annual Infections estimates by region

medRxiv preprint doi: https://doi.org/10.64898/2026.06.24.26356335; this version posted July 1, 2026. The copyright holder for this preprint (which was not certified by peer review) is the author/funder, who has granted medRxiv a license to display the preprint in perpetuity.
It is made available under a CC-BY 4.0 International license .

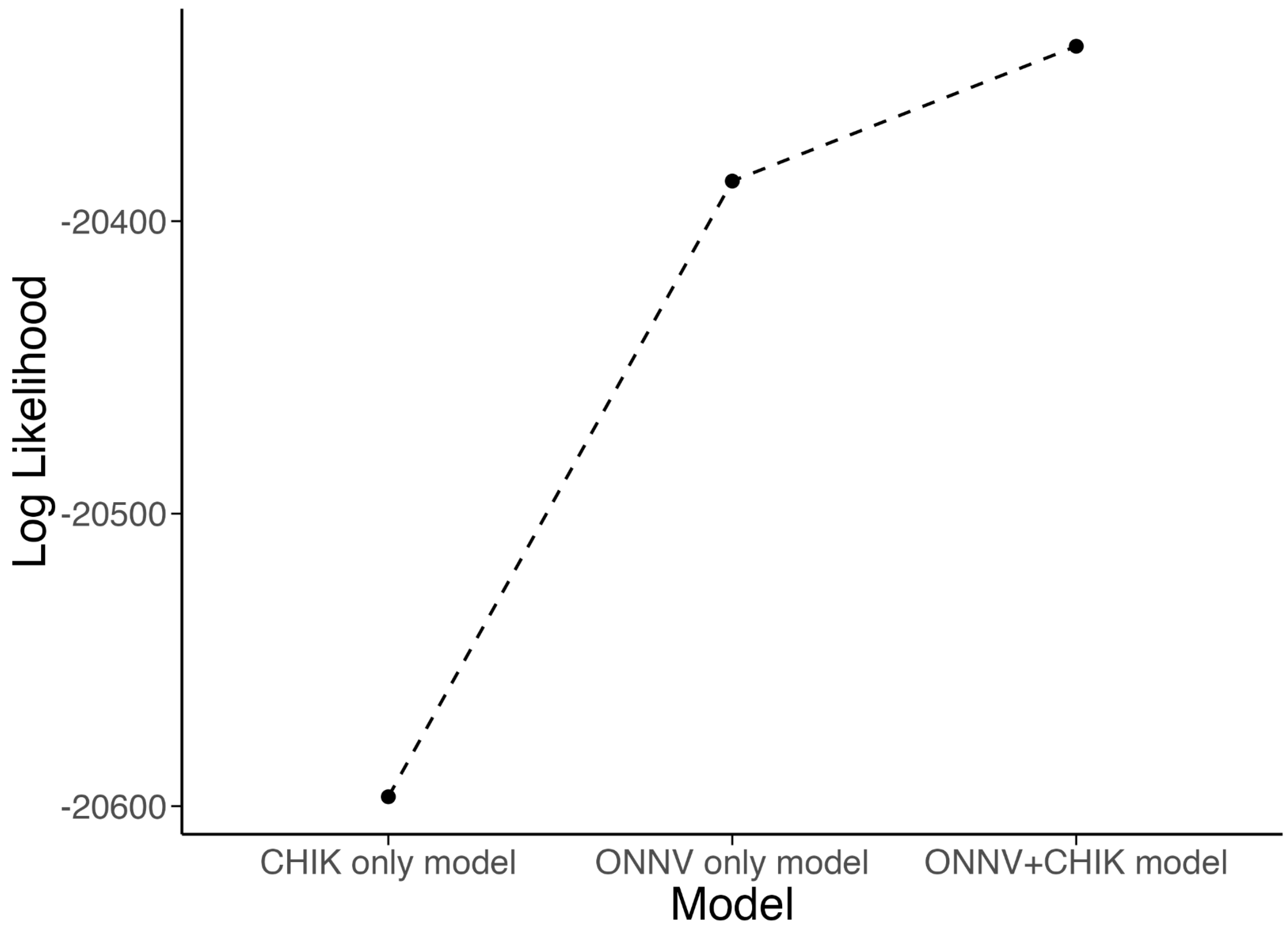

Supplementary Figure 1. Model fit comparison by log-likelihood across three models (CHIKV-only, ONNV-only, and ONNV+CHIKV). The combined ONNV+CHIKV model achieved the highest log-likelihood, supporting its selection as the final model.

medRxiv preprint doi: https://doi.org/10.64898/2026.06.24.26356335; this version posted July 1, 2026. The copyright holder for this preprint (which was not certified by peer review) is the author/funder, who has granted medRxiv a license to display the preprint in perpetuity.
It is made available under a CC-BY 4.0 International license .

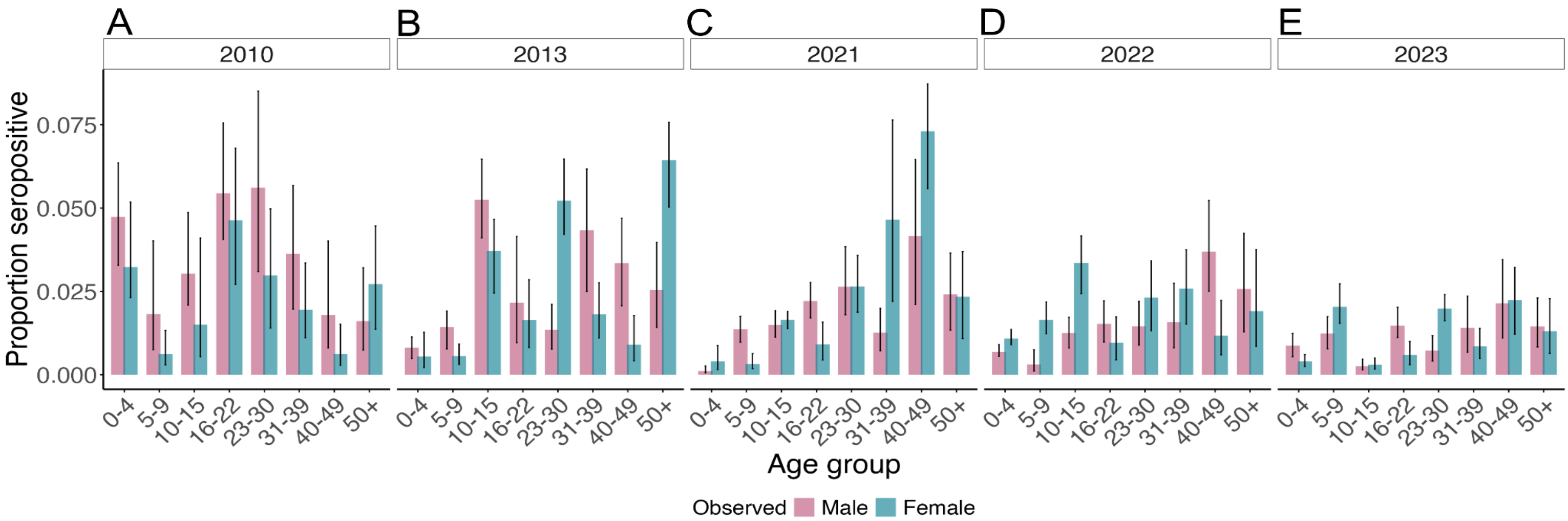

Supplementary Figure 2: CHIKV seropositivity by age and sex
(A–E) Proportion seropositive by age and sex for survey years 2010, 2013, 2021, 2022, and 2023, respectively. Bars represent median seroprevalence estimated by multivariate mixture model, with 95%CrI, stratified by gender.

medRxiv preprint doi: https://doi.org/10.64898/2026.06.24.26356335; this version posted July 1, 2026. The copyright holder for this preprint (which was not certified by peer review) is the author/funder, who has granted medRxiv a license to display the preprint in perpetuity.
It is made available under a CC-BY 4.0 International license .

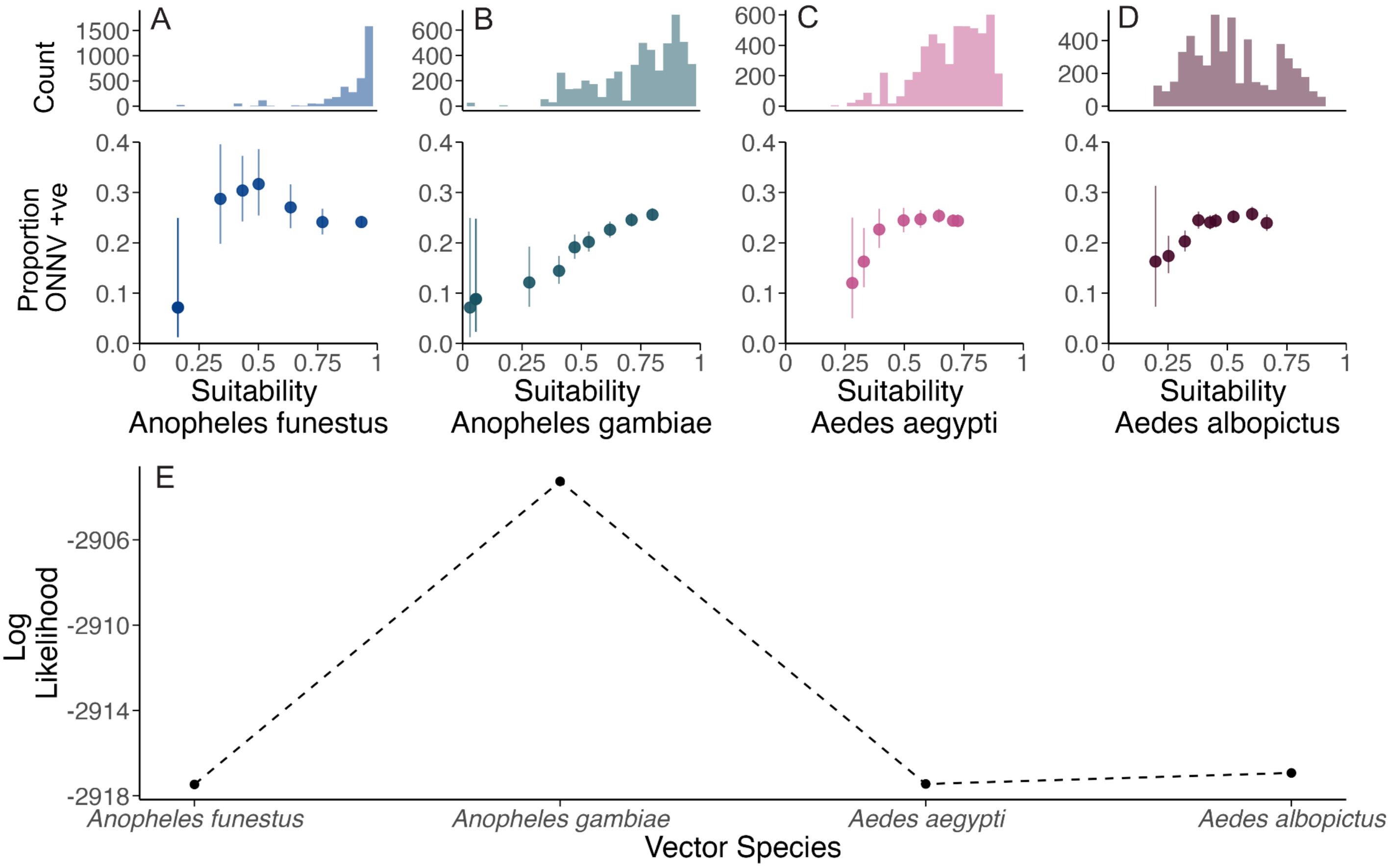

Supplementary Figure 3: **(A–D)** For each of four mosquito vectors: Anopheles funestus (blue), Anopheles gambiae (teal), Aedes aegypti (pink), and Aedes albopictus (mauve), upper panels show the frequency distribution of suitability across sampled locations, and lower panels show the relationship between ONNV seropositivity and vector suitability. **(E)** Log-likelihood comparison across four single-vector models, each incorporating the suitability of one candidate vector species as a covariate. Anopheles gambiae suitability yielded the highest log-likelihood, indicating the best model fit and supporting that Anopheles gambiae is associated with ONNV risk.

medRxiv preprint doi: https://doi.org/10.64898/2026.06.24.26356335; this version posted July 1, 2026. The copyright holder for this preprint (which was not certified by peer review) is the author/funder, who has granted medRxiv a license to display the preprint in perpetuity.
It is made available under a CC-BY 4.0 International license .

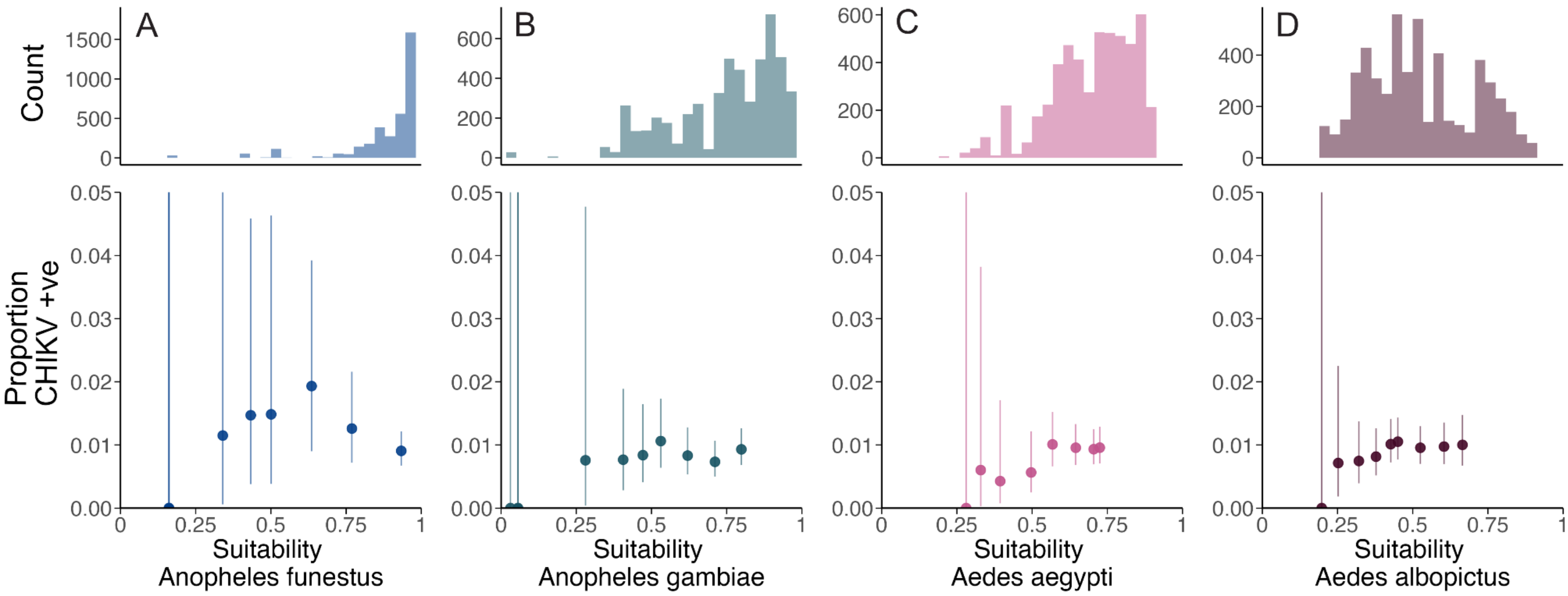

Supplementary Figure 4: **(A–D)** For each of four mosquito vectors: Anopheles funestus (blue), Anopheles gambiae (teal), Aedes aegypti (pink), and Aedes albopictus (mauve), upper panels show the frequency distribution of suitability across sampled locations, and lower panels show the relationship between CHIK seropositivity and vector suitability.

medRxiv preprint doi: https://doi.org/10.64898/2026.06.24.26356335; this version posted July 1, 2026. The copyright holder for this preprint
(which was not certified by peer review) is the author/funder, who has granted medRxiv a license to display the preprint in perpetuity.
It is made available under a CC-BY 4.0 International license .

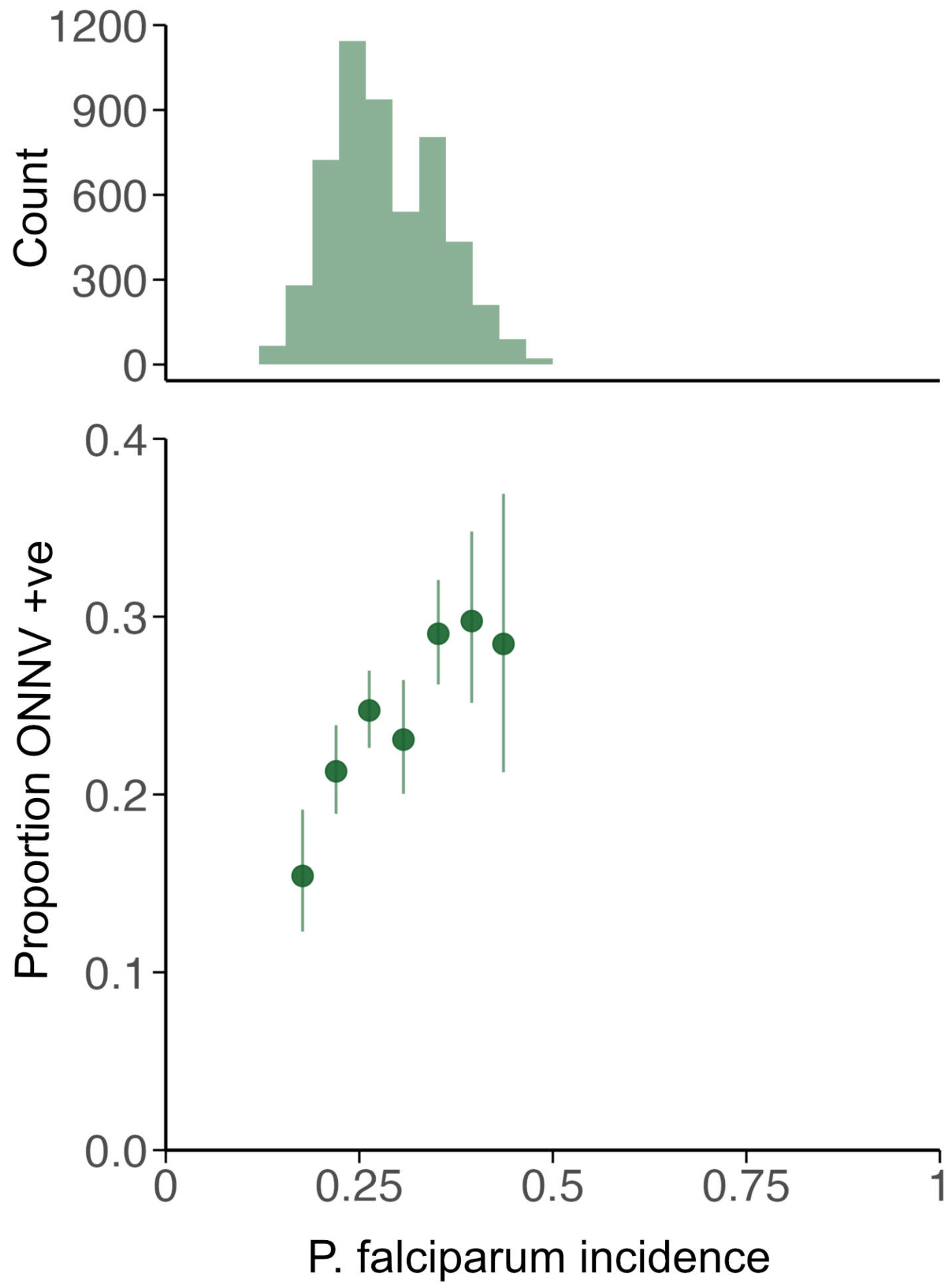

Supplementary Figure 5: Upper panel: frequency distribution of *P. falciparum* incidence across sampled locations. Lower panel: Relationship between ONNV seropositivity and *P. falciparum* incidence.
